# Winking earlobe sign

**DOI:** 10.1002/ccr3.5280

**Published:** 2022-01-11

**Authors:** Ryohei Ono, Togo Iwahana, Kaoruko Aoki, Hirotoshi Kato, Yoshio Kobayashi

**Affiliations:** ^1^ Department of Cardiovascular Medicine Chiba University Graduate School of Medicine Chiba Japan

**Keywords:** frank's sign, physical examination, pulse, tricuspid regurgitation, winking earlobe sign

## Abstract

The winking earlobe sign is a sign of tricuspid regurgitation, characterized by the movement of the earlobe coincident with the pulse.

## Graphical abstract

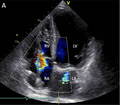

## 1 CLINICAL VIDEOS

**FIGURE 1:**
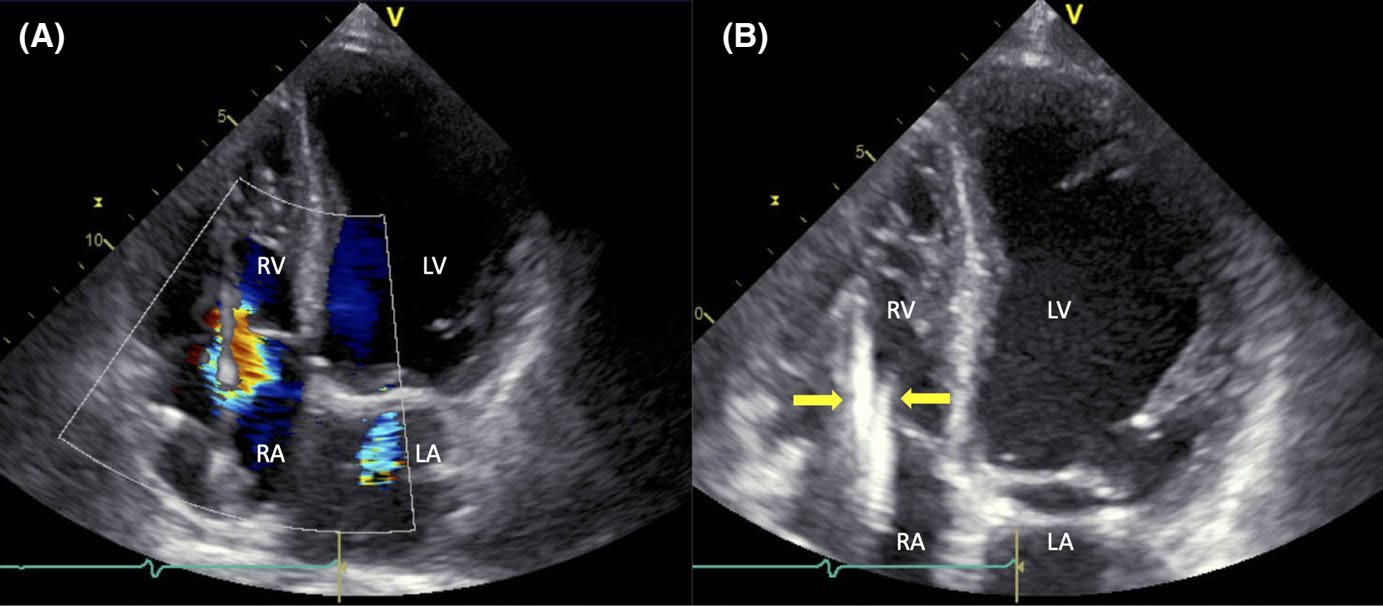
Transthoracic echocardiography showing severe tricuspid regurgitation (A) and two leads (arrows) entering the right ventricle through the tricuspid orifice (B). LA: left atrium, LV: left ventricle, RA: right atrium, RV: right ventricle

A 71‐year‐old man with a history of implantable cardioverter‐defibrillator (ICD) implantation presented with dyspnea. A physical examination showed Frank's sign, a diagonal crease in the earlobe, and remarkable systolic pulsations of the earlobe (winking earlobe sign) and the neck with jugular vein distension (Video S1). Moreover, a holosystolic murmur on auscultation and peripheral edema was noted. Transthoracic echocardiography revealed severe tricuspid regurgitation (TR) considered as ICD lead‐associated TR because two leads entered the right ventricle through the tricuspid orifice (Figure 1).

The winking earlobe sign is a sign of TR, characterized by the movement of the earlobe coincident with the pulse.^1^, ^2^

## CONFILCT OF INTEREST

None declared.

## AUTHOR CONTRIBUTIONS

RO contributed to patient management, conception, and design of case report; acquisition, analysis and interpretation of data, and drafting the article. TI, KA, and HK contributed to patient management, conception, and design of case report. YK contributed to interpretation of data and revised the article critically. All authors gave final approval of the article and have agreed to be accountable for all aspects of the work.

## ETHICAL APPROVAL

Written informed consent was obtained from the patient.

## CONSENT

Written patient consent has been signed and collected.

## Supporting information

Supplementary Material

Supplementary Material

## Data Availability

The data that support the findings of this study are available from the corresponding author upon reasonable request.
