# Supplementary material for: Winking earlobe sign
Source: Clin Case Rep. 2022 Jan 11;10(1):e05280. doi: 10.1002/ccr3.5280 (PMC8752453; doi:10.1002/ccr3.5280)
Supplement: Supplementary file 2 — Supplementary Material [file CCR3-10-e05280-s002.docx]

Video. Physical examination showing a pulsatile earlobe and pulsatile vessels at the base of the right neck.
